# Supplementary material for: A novel interpretable deep transfer learning combining diverse learnable parameters for improved T2D prediction based on single-cell gene regulatory networks
Source: Sci Rep. 2024 Feb 24;14:4491. doi: 10.1038/s41598-024-54923-y (PMC10891129; doi:10.1038/s41598-024-54923-y)
Supplement: Supplementary file 1 — Supplementary Figures. [file 41598_2024_54923_MOESM1_ESM.docx]

**Supplementary Figures**

| 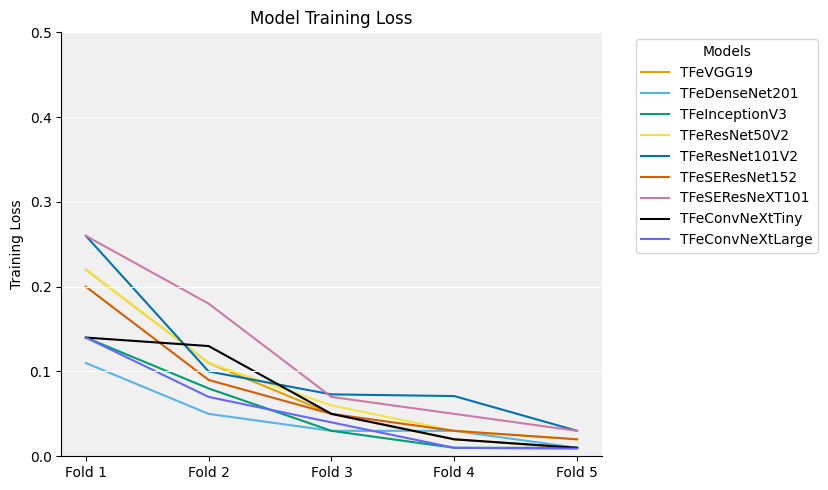  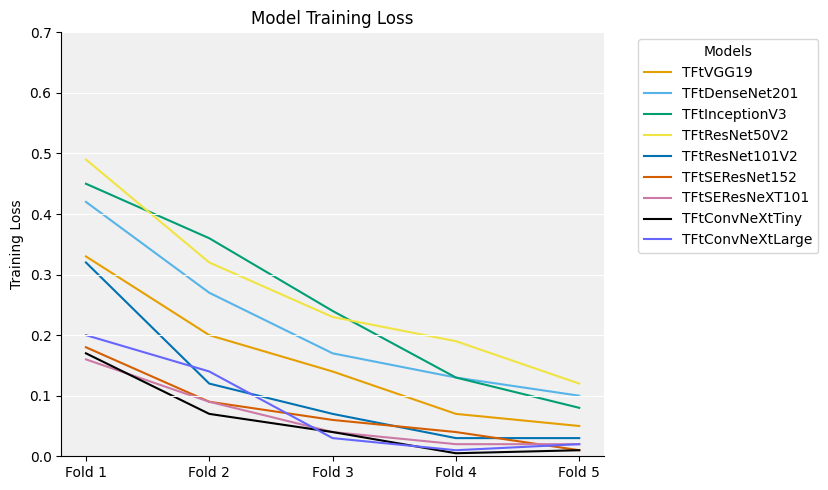  **Figure S1**: Reported training loss for epochs pertaining to TFe-based and TFt-based models when running five-fold cross-validation. TFe is transfer learning applying fine extraction with new classifier. TFt is transfer learning applying fine tuning with new classifier. |
| --- |

| 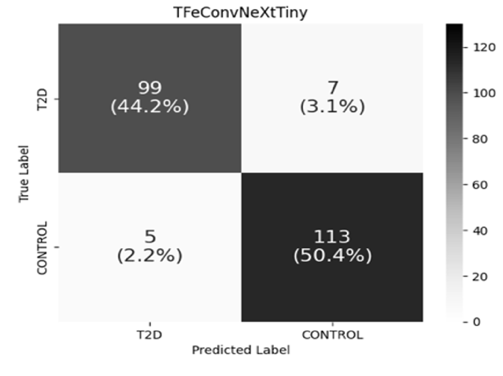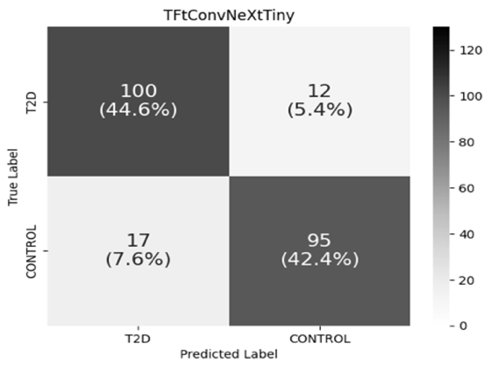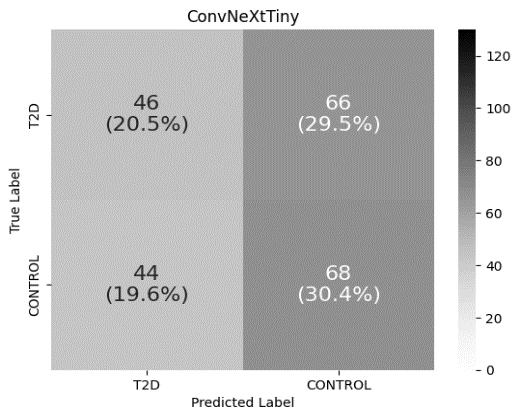  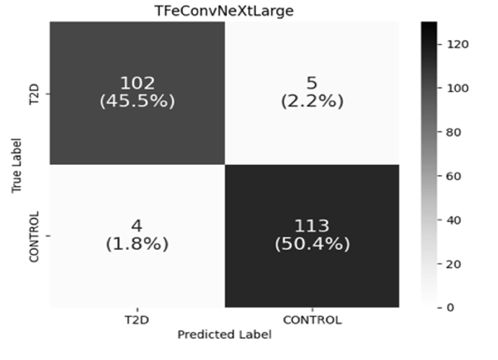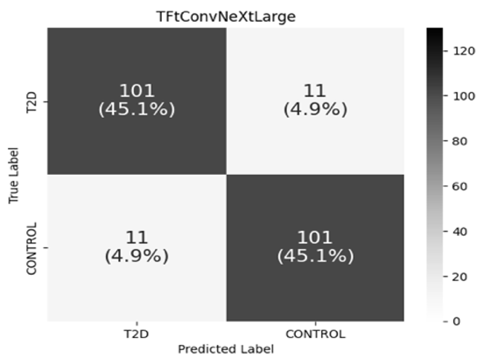  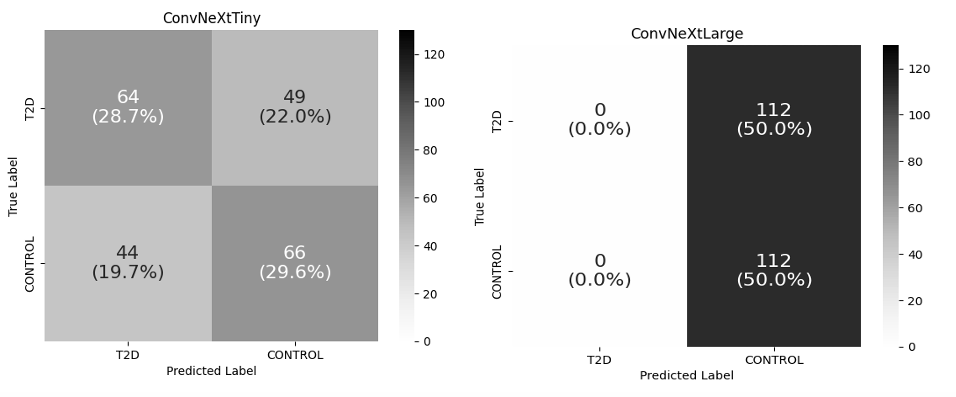  **Figure S2**: Combined confusion matrices for ConvNeXtTiny-based and ConvNeXtLarge-based models during the running of five-fold cross-validation. |
| --- |

| 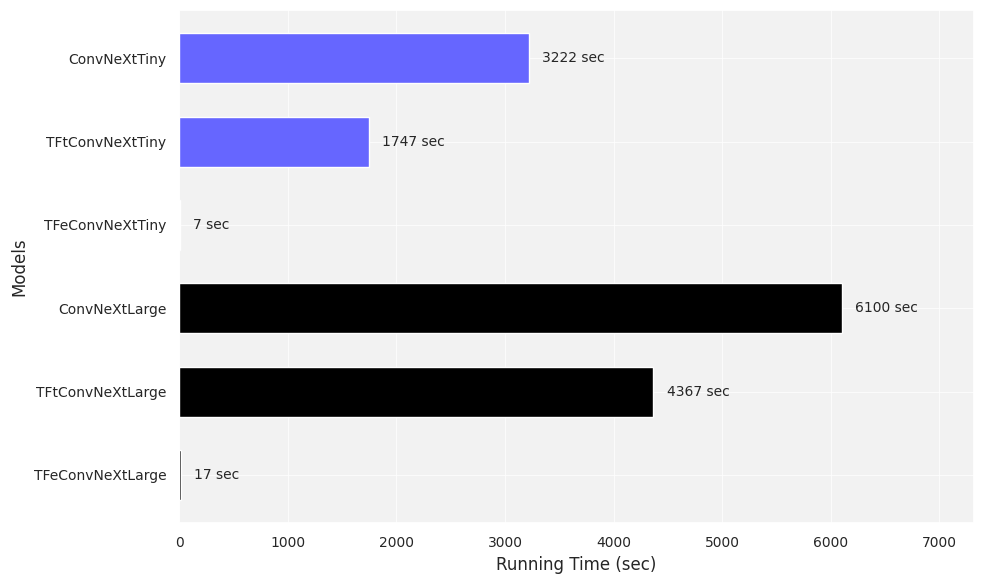 |
| --- |
| **Figure S3*:*** Running time comparisons in seconds for ConvNeXtTiny-based and ConvNeXtLarge-based models when running five-fold cross-validation. |
